# Automatic Construction and Global Optimization of a Multisentiment Lexicon

**DOI:** 10.1155/2016/2093406

**Published:** 2016-11-29

**Authors:** Xiaoping Yang, Zhongxia Zhang, Zhongqiu Zhang, Yuting Mo, Lianbei Li, Li Yu, Peican Zhu

**Affiliations:** ^1^School of Information, Renmin University of China, Beijing 100872, China; ^2^School of Computer Science, Northeastern University, Shenyang 110819, China; ^3^School of Computer Science and Technology, Northwestern Polytechnical University, Xi'an 710129, China

## Abstract

Manual annotation of sentiment lexicons costs too much labor and time, and it is also difficult to get accurate quantification of emotional intensity. Besides, the excessive emphasis on one specific field has greatly limited the applicability of domain sentiment lexicons (Wang et al., 2010). This paper implements statistical training for large-scale Chinese corpus through neural network language model and proposes an automatic method of constructing a multidimensional sentiment lexicon based on constraints of coordinate offset. In order to distinguish the sentiment polarities of those words which may express either positive or negative meanings in different contexts, we further present a sentiment disambiguation algorithm to increase the flexibility of our lexicon. Lastly, we present a global optimization framework that provides a unified way to combine several human-annotated resources for learning our 10-dimensional sentiment lexicon SentiRuc. Experiments show the superior performance of SentiRuc lexicon in category labeling test, intensity labeling test, and sentiment classification tasks. It is worth mentioning that, in intensity label test, SentiRuc outperforms the second place by 21 percent.

## 1. Introduction

Opinion mining and sentiment analysis of online text have become a hot research area in recent years, which includes opinion summarization and sentiment classification. Most of these tasks would benefit from a high quality sentiment lexicon which could provide excellent sentiment features when no training data is available.

The primary form of sentiment lexicons is binary annotation with positive and negative labels, such as Sentiwordnet developed by Italian Information Technology Research Institute [1, 2], the Chinese general sentiment lexicon (NTUSD) [3] annotated by Taiwan University, the Chinese emotion dictionary from the Chinese Academy of Sciences, and English Xsimilarity. Multiple sentiment lexicons with assignments of the strength of sentiments are also constructed, such as the Affective Lexicon Ontology of Dalian University of Technology (DUT Ontology) [4]. To determine the word-level strength of sentiment, manual methods, supervised methods employing WordNet or other semantic resources, and unsupervised approaches based on large-scale corpus were proposed. But few works evaluated and optimized the accuracy of intensity annotation by introducing all possible linguistic heuristics.

In recent years, driven by diverse tasks in different fields, both the polarity word and its related target are included as a sentiment item. However, the application areas of such 2-tuple lexicons as 〈polarity word, target〉 are strictly limited to one specific field, and also the size of such lexicons could easily explode with the growth of training data, which causes the problem of sparseness of features. Massive online text makes the limitations of domain sentiment lexicons increasingly apparent, especially when the sentiment classification tasks vary in different areas. Thus, a general and adaptable lexicon is important for sentiment analysis to avoid this problem.

This paper presents a method of automatic construction and optimization of a multisentiment lexicon through statistical analysis of a massive online corpus. The main content of this paper is as follows. First, we use neural network language model to obtain distributed representations of words from a massive online corpus (Sogou News Corpus, 3.17 GB) [5]. Second, we study the categorization of sentiments and select seed words for each category. After that, polarity words are selected and the semantic distances between polarity words and seed words are calculated with the distributed representations. The distance values are then converted into sentiment intensity through appropriate constraints. Finally, we evaluate the lexicon by combining linguistic heuristics in an optimization framework. Besides, we study sentiment tendency disambiguation method to improve the semantic description capability of our lexicon.

The remainder of this paper is organized as follows. In Section 2, we introduce some related works. The principle of automatic construction of SentiRuc is proposed in Section 3.  Section 4 introduces a unified optimization framework. Experiments and evaluations are reported in Section 5. We conclude the paper in Section 6 with future researches.

## 2. Related Work

Many Chinese sentiment lexicons, such as NTUSD, HowNet, and DUT Affective Lexicon Ontology, are manually annotated to ensure the lexicon's coverage and effectiveness. But manual methods usually cost too much labor and time and also tend to be subjective; the coverage is also a concern. To provide more granularities, it is necessary to introduce statistical language model to automatically annotate sentiment category and intensity.

To label the sentiments, we should first study the sentiment categorization. As early as 1957, Osgood distributed human emotion to three aspects: strong and weak, good and bad, active and passive [6]. In 2012, Liu et al. presented the DUT Affective Lexicon Ontology which contains 7 sentiments: happiness, liking, anger, sadness, hate, fear, and surprise [5]. QuanChangqin constructed Ren-CECps with 8 kinds of sentiments: expectation, joy, love, surprise, anxiety, sorrow, angry, and hate [7]. But existing categorizations of emotions are asymmetric. For example, there is no opposite emotion of “surprise” or “fear,” which can cause inconvenience in feature extraction and selection in supervised methods of sentiment analysis. Besides, there is coupling between emotion categories, such as “praise” and “like.” Therefore, sentiment classification needs to be investigated to suit both psychology and computational linguistics.

In addition to qualitative labeling, the sentiment intensity needs to be annotated quantitatively. A lot of the existing lexicons are manually annotated, including WordNet [8], General Inquirer [9], and HowNet [10]. To avoid the low efficiency and subjectivity of manual work, bootstrapping methods have been widely used. It is usually assumed that several seed words of known polarities are provided and different heuristics are adopted as the propagation strategy to infer the unknown sentiment polarities of other words. He sent the entries of HowNet into Google search and selected seed words according to the count of search results [11]. Li et al. introduced Pagerank to determine the polarity of words [12]. Each word is taken as a node in a graph, and HowNet is used to calculate the semantic similarity between seed words and candidate words as edge weights. The performance of these supervised methods is dependent on or limited by the accuracy of the third party tools or data. One possible way to solve this problem is to use unsupervised methods to obtain sentiment intensity from other corpuses or semantic resources. Colace et al. [13] construct the Mixed Graph of Terms by extracting 2-tuples like 〈side, evaluation〉 from comment text, and each item's intensity is inferred according to the domain knowledge in the Mixed Graph. Mukkamala et al. [14] define the expression of emotion as a fuzzy set of 4 elements 〈topic, keyword, object, and tendency〉. The relation strength of every 2 sets is determined through a membership function based on set theory and fuzzy logics. Turney and Littman [15] propose a semantic classification method based on emotional phrases. He first extracts the adjective or adverb phrases according to several defined templates and calculates the mutual information between words and phrases to determine the tendency and intensity of sentiment words. These unsupervised methods offered a lot of experience and help to us, but there is still much dependence on the accuracy of choosing, recognizing, and extracting various kinds of relationships of sentiment items.

Therefore, it is important to optimize the collection of sentiment lexicon entries and the intensity labeling. Chen et al. [16] construct polar square error function to decide if two entries have the same sentiment tendency and present an iterative expansion method. Turney and Littman [15] try to rationalize the intensity assignment by comparing the cooccurrence parameter of entries and seed words. Wang et al. [17] and Jo and Oh [18] both take tendency annotation as a by-product of sentiment classification task and yet failed to evaluate the annotation's quality. Some scholars try to introduce synonymous or antonymous relationships into the evaluation framework to optimize the intensity labeling [19, 20]. Compared with our work, the optimization framework of mentioned works is relatively simple and fails to take multisentiment words into consideration, which may express different tendency in various contexts.

Considering the above points, this paper presents an unsupervised model of automatic construction of a multisentiment lexicon based on WLI neural network language model [21] and a global optimization framework. The main contributions of this paper are as follows:

1. We propose a new categorization of human emotions, which makes the linguistic features more suitable for computational analysis.
2. We define the converting constraint set of distance and sentiment intensity and present an automatic construction model based on WLI language model.
3. We present a global optimization framework based on several manually annotated semantic resources, to improve the semantic description of our lexicon SentiRuc.

## 3. Automatic Construction of SentiRuc

In this section, we present the “5 pairs with 10 polarities” categorization of human emotions and automatically annotate the multisentiment lexicon SentiRuc by defining the converting constraint set of distance and sentiment intensity. We also investigate the emotional disambiguation of multiple affective words in this section.

We integrated the entries of NTUSD dictionary, HowNet lexicon, and the DUT Ontology as the entries of our SentiRuc lexicon, which contains a total of 14250 emotional words.

### 3.1. WLI Language Model and the Categorization of Human Emotions

Traditional binary sentiment labeling has gradually become unable to meet the development of sentiment analysis tasks. The primary work of multiple sentiment labeling is the categorization of human emotions. Section 2 has discussed relevant achievements and existing problems. This paper takes the achievement of psychology, linguistics theory, and computation characteristics into consideration and categorizes human emotions to 10 categories: happy-sad, like-hate, believable-unexpected, gratitude-angry, and complementary-critical. Each pair contains 2 opposite sentiment polarities. Our goal is to annotate each sentiment word W with a 10-dimensional sentiment vector Senti(W), and the value of each dimension represents the intensity of the corresponding sentiment tendency. In the following research, the 10 words are directly adopted as seed words.

Words contain very rich meanings, and statistical language models are used to extract those semantic features. Given a corpus, neural network language model could map words into a high dimensional continuous space. Word2Vec is a tool based on deep learning and is released by Google in 2013, which adopts two main language models: the continuous bag of words model and continuous skip-gram model [22]. Mikolov et al. also found that the representations have very good linear semantic characteristics [23], so, in 2015, WLI neural network language model is presented to decrease the model complexity [21]. We accumulate the offset of corresponding dimensions of two-word representations as the linear semantic distance between them and further investigate how coordinate offset could affect word similarity. In this paper, we use Sogou News Corpus as the training set, which contains about 1.1 million different words.

### 3.2. Converting the Distances of Words into Word Similarities

All word representations are located in a high dimensional vector space, in which we determine an entry's polarity and intensity by computing the distance between the entry and seed words. However, there are many words that could express, for example, happiness. And it is difficult to choose one as the only seed of “happy.” Here, to decrease the deviation caused by subjectivity, we use coordinate offset of word representations to list the 50 nearest neighbors of “happy” and then manually choose several words as the seed set of “happy.” For example, we collect all distances between “bittersweet” and “happy” seeds and take the average distance as the distance between “bittersweet” and “happy” emotion. For any word W, we can obtain a 10-dimensional distance vector Dis(W) and each dimension of Dis(W), respectively, represents the distance between W and happy, like, believable, gratitude, complimentary, sad, hate, unexpected, angry, and critical

(1) Disbittersweet=1.13,3.09,3.70,2.08,4.45,1.34,4.14,2.56,5.41,2.72.

Previous research pointed out that, generally, a word mainly contains only one or two emotions [5], so we preserve the minimum one or two distances in Dis(W) as effective distances. Larger distances will be abandoned, which means that those sentiments with lower similarities will be eliminated. If threshold *T* is assigned with 3.00 for the word “bittersweet,” only 2 distances in Dis(W), happy 1.13 and sad 1.34, will be retained, because the sum of the 2 distances has not reached *T*. That could be interpreted as “happy” and “sad” being the main sentiments contained in “bittersweet.” Only these two distances are retained and used in the followup work and only these 2 distances are retained and used as “effective distances” in the followup work.

Paper [23] points out that linear coordinate offset between word representations is directly associated with words' semantic similarities. Therefore, we could annotate a word W's polarity intensity according to the coordinate offset between W and seed words in the vector space of word representations. Considering there could be more than one effective distances in Dis(W), it is necessary to investigate how different distributions of those distances impact words' similarities. To solve the problem of converting distance vector Dis(W) to sentiment vector Senti(W), we define 3 converting constraints.

**Constraint 1 (diversity constraint).**

Each dimension of Senti(W) is denoted as Senti(W)[*i*] (*i* is an integer ranging from 1 to 10), indicating word W's intensity of each sentiment category. Senti(W)[*i*] shall be negatively correlated with the count of effective distances Count(Dis(W)), because it is observed that words with more effective distances usually lie farther away from each sentiment category, which could be interpreted as the sentiment intensities being “distracted” by various polarities. For example, “rage” is only 1.92 away from the “angry” category, while “unfair” is 3.38 away from “angry” and 5.05 away from “critical.”

**Constraint 2 (self constraint).**

Each dimension of Dis(W) is denoted as Dis(W)[*i*] (*i* is an integer ranging from 1 to 10), indicating the distance between word W and each sentiment category. The intensity of a certain sentiment Senti(W)[*i*] shall be negatively correlated with the corresponding distance Dis(W)[*i*]. The fact is that, in word representations, smaller distance indicates more semantic or pragmatic similarities.

**Constraint 3 (global contrast constraint).**

The intensity of a certain sentiment Senti(W)[*i*] shall be negatively correlated with the ratio of Dis(W)[*i*] and the average effective distance Avg(Dis(W)). In a language, human habits cause big difference in word frequencies, and collocation of words also divides words into various clusters. These both impact the quantized word representations. For example, the effective distance vector of “enjoy” is (2.09, 1.11, 0, 0, 0, 0, 0, 0, 0, 0) and that of “enchanted” is (5.26, 3.87, 0, 0, 0, 0, 0, 0, 0, 0). The global contrast constraint is used to eliminate this disparity.

From the converting constraints set we could derive the generating formula of W's sentiment vector Senti(W) as follows:

(2) $$\begin{array}{c} Senti\left(\;W\;\right)\left[\;i\;\right]=\text{Diverge}\cdot \text{Self}\cdot \text{Contrast}. \end{array}$$

Senti(W)[*i*] indicates word W's intensity of each sentiment category. The formula contains three factors: the factor of diversity constraint Diverge, the factor of self constraint Self, and the factor of global contrast constraint Contrast. These factors can be, respectively, expressed as follows:

(3) DivergeDisW=C0α·CountDisW+C1,

(4) SelfDisW=C2DisWi+C2β,

(5) ContrastDisW=AvgDisWDisWiγ.

In formulas (3), (4), and (5), Count(Dis(W)) represents the count of effective distances and Avg(Dis(W)) is the average value of effective distances. According to constraints 1, 2, and 3, the positive or negative correlation has already been illustrated by denominators or numerators in formulas (3), (4), and (5). *C*_0_, *C*_1_, and *C*_2_ are constants. In the experiments in Section 5, we will introduce the assignments of *C*_0_, *C*_1_, and *C*_2_. The 3 parameters *α*, *β*, and *γ*,respectively determine the effect of each constraint. The optimal parameters can be trained through the optimization framework (Section 4).

Finally, to every sentiment word W, we annotate it in our sentiment lexicon with a 10-dimensional vector Senti(W). The value in each dimension represents the similarity between W and this sentiment, that is, W's intensity of this sentiment.

### 3.3. Sentiment Tendency Disambiguation Based on Word Distribution Density

In Section 3 we introduced an automatic method to identify a word's polarity and intensity. But some words convey different sentiment polarities in different contexts. It would be inappropriate to annotate such words in only one sentiment vector, so we investigate sentiment disambiguation in this section. Chen et al. [24] pointed out that “sentiment disambiguation is different from word sense disambiguation” because, in a general sentiment lexicon, a word's sentiment tendency is not directly correlated with its meaning.

We use a hybrid approach in screening multisentiment words from our lexicon's vocabulary. So far there is no effective method for automatic selection of multisentiment words. We attempted to extract words which appear in different synonym sets in “HIT Tongyicicilin” and “Synonym Lexicon for Pupils” and take these words as the candidate set S of multisentiment words. However, S shows good precision but poor recall. For example, “naive” could convey both positive and negative senses but is not covered in the candidate set. We finally decide to manually select multisentiment words from “HIT Tongyicicilin” and “Synonym Lexicon for Pupils” and take these words as multisentiment word set S_MultiSenti_ which include altogether 148 entries.

Then, 113694 sentences containing words in S_MultiSenti_ are selected from Sogou News Corpus, and the sentiment tendency of these words is annotated with a positive or negative label. In a context window size of 16, the distribution density of each context word is extracted and used as a feature of SVM classifier. The distribution density of a context word CW can be obtained by

(6) ρCWpositive=CountCWpositiveCountCW,ρCWnegative=CountCWnegativeCountCW.

Count(CWpositive) represents the count of context word CW in all sentences which have a positive W. Count(CWnegative) is the count of CW in all sentences that have a negative W. Count(CW) is the total count of CW in all sentences where W appears.

After the tendency disambiguation, a multisentiment word W is split and segmented as two independent cases W_positive_ and W_negative_. The word representations would then be trained again, and the sentiment vectors of W_positive_ and W_negative_ could be generated through formula (2).

## 4. Global Optimization Framework

Section 3 presented a converting constraint set, and our lexicon SentiRuc is preliminarily generated. This section establishes a unified form of evaluation function to study the effects of various constraints. We've collected data from HIT Tongyicicilin, Synonym Lexicon for Pupils, Antonym Lexicon for Pupils, and the dataset of NLPCC 2013 Competition and NLPCC 2014 Competition. These datasets are all manually constructed resources and thus could be regarded as gold standards. The error function is used to evaluate the deviation between SentiRuc and those gold standards, and our goal is to find a set of parameters that minimizes the deviation.

### 4.1. Synonymous Relationship

If W_1_ and W_2_ are annotated as a pair of synonyms in HIT Tongyicicilin or Synonym Lexicon for Pupils, we can infer that their sentiment polarities and intensities tend to be similar. To formalize this intuition, we accumulate the deviation of sentiment intensity of W_1_ and W_2_ on corresponding dimensions in SentiRuc. The error function is shown as follows:

(7) $$\begin{array}{c} f_{1}\left(\;D\;\right)=\frac{1}{\left|D_{1}\right|}\underset{D_{1},i}{\sum }\frac{Senti\left(W_{1}\right)\left[i\right]-Senti\left(W_{2}\right)\left[i\right]}{Senti\left(W_{1}\right)\left[i\right]+Senti\left(W_{2}\right)\left[i\right]}. \end{array}$$

Senti(W)[*i*] indicates word W's intensity of each sentiment category. In formula (7), W_1_ and W_2_ are labeled as synonyms and Pair(W_1_, W_2_) ∈ *D*_1_, |*D*_1_| is the count of synonym pairs. *f*_1_(*D*) represents the average deviation of sentiment intensity of W_1_ and W_2_ on corresponding dimensions, when W_1_ and W_2_ are in both SentiRuc and synonym resources. *f*_1_(*D*) varies with parameters *α*, *β* and *γ* in Formulas (3), (4), and (5).

### 4.2. Antonymous Relationship

If W_1_ and W_2_ are annotated as a pair of antonyms in Antonym Lexicon for Pupils, we can infer that they shall have opposite sentiment polarities, where the intensities tend to be similar. In accordance with this intuition, we accumulate the deviation of sentiment intensity of W_1_ and W_2_ on opposite dimensions in SentiRuc. The error function is shown as formula (8)

(8) $$\begin{array}{c} f_{2}\left(\;D\;\right)=\frac{1}{\left|D_{2}\right|}\underset{D_{2},i,j}{\sum }\frac{Senti\left(W_{1}\right)\left[i\right]-Senti\left(W_{2}\right)\left[j\right]}{Senti\left(W_{1}\right)\left[i\right]+Senti\left(W_{2}\right)\left[j\right]}. \end{array}$$

Senti(W)[*i*] indicates word W's intensity of each sentiment category. In formula (8), W_1_ and W_2_ are labeled as antonyms (Pair(W_1_, W_2_) ∈ *D*_2_), and *i*, *j* are the indices of opposite sentiments in sentiment vectors of W_1_ and W_2_. |*D*_2_| is the count of antonym pairs. *f*_2_(*D*) represents the average deviation of sentiment intensity of W_1_ and W_2_ on opposite dimensions, when W_1_ and W_2_ are in both SentiRuc and antonym resources. *f*_2_(*D*) varies with parameters *α*, *β*, and *γ* in formulas (3), (4), and (5).

### 4.3. Sentiment Ratings at the Sentence Level

If the annotation of SentiRuc could contribute more in relevant tasks, the sentiment classification result using SentiRuc shall be closer to human judgment than that using other lexicons. We select 6000 sentences from the dataset of NLPCC 2013 Competition and NLPCC 2014 Competition and label the sentences with a “main sentiment” and an optional “subsentiment,” which are both involved in the 10 sentiment categories of SentiRuc. For a certain set of parameters in formulas (3), (4), and (5), we generate an individual annotation of SentiRuc for the sentiment classification task. The sentence-level error function is constructed by Jaccard similarity of classification result and labeled result, represented as follows:

(9) $$\begin{array}{c} f_{3}\left(\;D\;\right)=\frac{1}{\left|D_{3}\right|}\underset{D_{3}}{\sum }\text{Jaccard}\left(\;\text{Label}\left(\;d_{i}\;\right),\text{Sentence}\left(\;d_{i}\;\right)\;\right). \end{array}$$

*d*_*i*_ is the identifier of each sample. |*D*_3_| is the number of sentences contained in the dataset. Label(*d*_*i*_) represents the labeled sentiment vector of a sentence and Sentence(*d*_*i*_) is the classified sentiment vector using SentiRuc. *f*_3_(*D*) shows the average of Jaccard similarity of each sentence's labeled result and classification result. *f*_3_(*D*) varies with parameters *α*, *β*, and *γ*.

### 4.4. The Global Error Function

**Figure 1:**
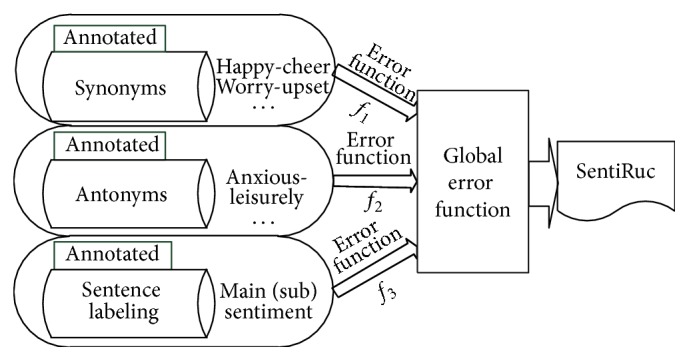
The global optimization framework.

By combining the above three evaluation methods we obtain the global optimization framework based on manually constructed resources, as shown in Figure 1.

The global error function is

(10) $$\begin{array}{c} f\left(\;D\;\right)=f_{1}\left(\;D\;\right)+f_{2}\left(\;D\;\right)+f_{3}\left(\;D\;\right). \end{array}$$

The global error *f*(*D*) varies with parameters *α*, *β*, and *γ* in formulas (3), (4), and (5). By minimizing *f*(*D*) we can find the optimal parameter set argmin⁡(*f*(*D*)).

## 5. Experiments

We first evaluate the generating process of SentiRuc and then verify the availability of SentiRuc. To evaluate the rationality of the generating process, we design the parameters tuning experiment to prove the rationality of constraint set (Section 5.1) and verify the validity of sentiment tendency disambiguation method (Section 5.2). To test the availability of SentiRuc, we compare the qualitative and quantitative annotation of SentiRuc with other lexicons (Section 5.3) and investigate the performance of different lexicons in sentiment classification tasks (Section 5.4). NTUSD Lexicon of Taiwan University, HowNet sentiment lexicon, and DUT Ontology are all involved in the experiments.

In all experiments, the threshold distance value *T* is assigned with Avg(Dis(W)) so that only one or two kinds of sentiments would remain for each word. The parameter *C*_0_ in formula (3) is set to 10, representing the number of sentiment categories. *C*_1_ is set to 8, which means the number of sentiment categories minus the maximum remaining sentiments (10 − 2 = 8). *C*_2_ is set to 3.38, representing the average coordinate offset between every two words included in SentiRuc. The dimension number of word representations is 60. Either more or less dimensions would increase the value of error function of the intensity annotating result.

Sogou News Corpus (3.17 GB) is used as the training text set. After segmentation by ICTCLAS 5.0 developed by Chinese Academy of Sciences, this corpus contains about 0.83 billion words, and the vocabulary size is 1,104,914. We do not have any other preprocessing of the data, so it can be ensured that every n-gram sample is a real Chinese word sequence and also that the word representations can show the actual semantic distribution of each word.

### 5.1. Evaluation of the Generating Constraint Set

Section 3 introduced how to automatically annotate the multisentiment lexicon SentiRuc by defining the converting constraint set of distance and sentiment intensity.  Section 4 presented a global optimization framework to optimize the parameters. We first set *α*, *β*, and *γ* to 1 as the baseline experiment. If a parameter is set to zero, it can be regarded as if this parameter is ignored. We conduct some contrast experiments where one parameter is dropped out in each experiment. The experimental result of *f*(*D*) with the parameters is shown in Table 1.

It can be seen that dropping any constraint would increase the global error, which indicates that all constraints are useful in computing the intensity of SentiRuc. When *β* is dropped, *f*(*D*) increases the most, which suggests that the self constraint contributes the most. It means that the intensity of a certain sentiment is remarkably negatively correlated with the corresponding distance, which proves the rationality of our method based on word representations. Then we try to find the optimal parameter set through variable-controlling method. As shown in the bottom three rows, the global error is further decreased, and the optimal parameter set is listed in the bottom line.

### 5.2. Evaluation of Tendency Disambiguation

Section 3.3 introduced how the 148 multisentiment words are selected. From Sogou News Corpus we collect sentences which contain these multisentiment words and label a multisentiment word W with “1” when W expresses positive tendency and with “2” if W contains negative tendency. To ensure excellent label result, eight Chinese native speakers participated in the annotating work. Every researcher made independent annotation of about 50 thousand sentences and each sentence is annotated by 4 researchers. If there is confliction in a sentence's label result, we made the final result through a panel discussion. In total, 113,694 sentences are annotated with positive tag or negative tag.

According to the labeled result, the tendency disambiguation algorithm based on word distribution density, which is introduced in Section 3.3, is used in the experiment. The experimental results of tenfold cross validation are shown in Table 2.

The overall disambiguation accuracy of all 148 words in the 113,694 sentences reaches 95.52%. The entries “epigone” and “yes-man” get the lowest accuracy, mainly due to the limited training data caused by their low occurrence. Generally, this experiment result shows that our disambiguation algorithm can effectively distinguish different tendencies of a word.

### 5.3. Evaluation of the Annotation's Quality of Sentiruc

The sentiment polarity and intensity of SentiRuc are both drawn from a Chinese corpus of GB grade level; therefore, its semantic description should be closer to actual semantic distribution than manually constructed lexicons. We try to evaluate the annotation quality of several existing lexicons by analyzing their sentiment category consistency (qualitative evaluation) and sentiment intensity consistency (quantitative evaluation). Sentiment category consistency examines the similarity of synonyms' (or antonyms') tendency annotation in SentiRuc. Sentiment intensity consistency refers to the similarity of synonyms' (or antonyms') intensity annotation in SentiRuc.

HIT Tongyicicilin and Synonym Lexicon for Pupils contain 55,265 synonyms, from which we selected 2500 synonyms as the test dataset S_syn2500_. The 1774 antonyms in Antonym Lexicon for Pupils are taken as test dataset S_ant1774_. Multisentiment words are not included in S_syn2500_ or S_ant1774_. The intensity consistency Value_synonym_ and intensity consistency Value_antonym_ can be expressed as

(11) Valuesynonym=1−1Dsame∑SentiW1i−SentiW2iSentiW1i+SentiW2i,Valueantonym=1−1Doppo∑SentiW1i−SentiW2i+5SentiW1i+SentiW2i+5.

Senti(W)[*i*] (*i* is an integer ranging from 1 to 5) indicates word W's intensity of each positive sentiment category. *D*_same_ and *D*_oppo_ represent the count of corresponding dimensions which are annotated with nonzero value.

The evaluation result of synonyms is in Table 3 and that of antonyms is shown in Table 4.

Tables 3 and 4 indicate that, in SentiRuc, the tendency annotation of synonyms and antonyms are both closer to manually labeled resources than other sentiment lexicons. In the condition that each word's sentiment intensity is computed independently, the intensity consistency of synonyms and antonyms in SentiRuc reaches 92% and 91%. This score is up to 20 percentage points higher than the manual intensity annotation of DUT Ontology, which is far more than expected. The results prove the effectiveness of the converting constraint set and formula (2) and also indicate that SentiRuc has better semantic descriptiveness.

### 5.4. Evaluation of SentiRuc in Sentiment Analysis Tasks

This experiment investigates the performance of sentiment analysis task using different lexicons. 3,100 sentences are selected from NLPCC 2013 Competition and NLPCC 2014 Competition and 3,700 sentences containing one of the 148 multisentiment words are selected from Sina Microblog. All 6,800 sentences are labeled with a “main sentiment” and an optional “subsentiment” tag. We define 2-gram part of speech (2-POS) and 3-gram part of speech (3-POS) for every labeled sample and extract sentiment tendency features with the help of SentiRuc. SVM is used in the multivariate classification experiments. Compared with human annotation result, the accuracy of the multivariate classification reaches 62.0%.

In order to facilitate the comparison of different lexicons, we also conduct binary classification experiments (positive or negative). Each of the 6,800 sentences is labeled with a “positive” or “negative” tag by four Chinese native speakers. The other 3200 objective sentences without affection are also labeled with “neutral” and added in the test dataset. For each sentence, we extract the 2-POS and 3-POS features and identify sentiment features with the help of SentiRuc. We use SVM classifier to implement tenfold cross validation. In addition, we also investigate the performance of SentiRuc before and after the tendency disambiguation. The results can be evaluated by

(12) Precision=Result_CorrectResult_Proposed×100%,Recall=Result_CorrectResult_Labeled×100%,F-measure=2×Precision×RecallPrecision+Recall×100%.

Result_Correct is the number of sentences that are correctly labeled with “positive” (or “negative”). Result_Proposed is the number of sentences labeled with “positive” (or “negative”) by SVM model. Result_Labeled is the number of sentences manually labeled with “positive” (or “negative”). The result is shown in Table 5.

Table 5 indicates that the* F*-measure of positive and negative classification using SentiRuc is apparently higher than those using other lexicons. In all 6800 subjective sentences, the sentences containing multisentiment words account for 54.4% and such a high percentage results in an apparent difference before and after disambiguation. Such a high percentage also brings impact on the overall* F*-measure on general domain text, respectively, 0.726 and 0.627. Actually, on the 6300 sentences which do not contain any multisentiment word, the* F*-measure of positive text and negative text is, respectively, 0.817 and 0.742.

## 6. Conclusion

This paper presented an automatic construction and global optimization framework of a multisentiment lexicon SentiRuc. The main jobs include the categorization of human emotions, an automatic construction model based on WLI language model, a global optimization framework based on several manually annotated semantic resources, and the disambiguation of multisentiment words. The experiment in Section 5 indicates that SentiRuc performs well on general dataset. Particularly, in intensity labeling test, SentiRuc outperforms the second place by 21 percent, which proves that statistical language modeling performs outstandingly in the semantic representation of sentiments. Our lexicon is now available online (https://pan.baidu.com/s/1jHAInlG).

It is difficult to directly compare existing lexicons because of various sentiment categorizations. We will investigate appropriate evaluation method of multiclass sentiment classification tasks.

Although Section 5 has shown the outstanding performance of word representations in sentiment lexicon's construction, the unique features of word representations still bring problems to text mining tasks. Firstly, statistical language models depend a lot on the correspondence of inner semantics and outer grammars; thus, it is of great significance to research how to comprehend and distinguish “similar” words, “related” words, and their association with word representations' generating models. Secondly, similar words' vectors only differ much at several specific dimensions and further research on this kind of characterization is needed. We will study weighted statistical language models and will investigate the feasibility and effect of introducing various vector operations into the estimation of semantic distances.

## Figures and Tables

**Table 1:** The tuning of generating parameters.

	*α*	*β*	*γ*	*f*(*D*)
Baseline	1	1	1	2.301

Dropping one constraint	0	1	1	2.402
	1	0	1	2.660
	1	1	0	2.599

Parameter tuning	1.875	1	1	2.123
	1.875	1.075	1	2.046
	1.875	1.075	1.145	1.972

**Table 2:** Experiments of sentiment disambiguation.

	Target	Samples	Accuracy
Overall	148 words	113694	95.52%
Highest accuracy	滋生 breed	3095	98.71%
	*幼稚* naive	1924	98.70%
Lowest accuracy	*息事宁*人 yes-man	281	87.90%
	*萧规曹随* epigone	130	61.54%

**Table 3:** Evaluation of synonyms in each lexicon.

Lexicons	Synonyms	Category consistency	Intensity consistency
NTUSD	2179	87.29%	—
HowNet	2500	89.04%	—
DUT	2500	88.44%	70.89%
SentiRuc	2500	91.88%	92.54 %

**Table 4:** Evaluation of antonyms in each lexicon.

Lexicons	Antonyms	Category consistency	Intensity consistency
NTUSD	1450	84.00%	—
HowNet	1772	86.51%	—
DUT	1774	85.40%	67.55%
SentiRuc	1774	87.94%	91.62 %

**Table 5:** Sentiment classification based on different lexicons.

Result of positive text			
Lexicon	Precision	Recall	*F*1
NTUSD	0.603	0.375	0.462
HowNet	0.728	0.540	0.620
DUT	0.721	0.552	0.593
SentiRuc (before disambiguation)	0.744	0.588	0.657
SentiRuc (after disambiguation)	0.782	0.678	0.726

Result of negative text			
Lexicon	Precision	Recall	*F*1

NTUSD	0.480	0.319	0.383
HowNet	0.611	0.451	0.519
DUT	0.572	0.445	0.501
SentiRuc (before disambiguation)	0.633	0.468	0.538
SentiRuc (after disambiguation)	0.671	0.589	0.627
